# Intraabdominal sporadic desmoid tumors and inflammation: an updated literature review and presentation and insights on pathogenesis of synchronous sporadic mesenteric desmoid tumors occurring after surgery for necrotizing pancreatitis

**DOI:** 10.1007/s10238-022-00849-6

**Published:** 2022-08-01

**Authors:** Francesco Prete, MariaTeresa Rotelli, Alessandro Stella, Giovanna Calculli, Lucia Ilaria Sgaramella, Antonio Amati, Nicoletta Resta, Mario Testini, Angela Gurrado

**Affiliations:** 1 Academic General Surgery Unit, Department of Biomedical Sciences and Human Oncology, University of Bari “Aldo Moro” Medical School, 11, Piazza Giulio Cesare, 70124 Bari, Italy; 2 General Surgery and Liver Transplantation Unit, Department of Emergency and Organ Transplantation, University of Bari “Aldo Moro” Medical School, Bari, Italy; 3 Division of Medical Genetics, Department of Biomedical Sciences and Human Oncology, University of Bari “Aldo Moro” Medical School, Bari, Italy; 4 Division of Pathology, Department of Emergency and Organ Transplantation, University of Bari “Aldo Moro” Medical School, Bari, Italy

**Keywords:** Desmoid tumor, Inflammation, Pancreatitis, Beta catenin, Mutation, microRNA

## Abstract

Sporadic intra-abdominal desmoid tumors are rare and known to potentially occur after trauma including previous surgery, although knowledge of the underlying pathogenetic mechanism is still limited. We reviewed the recent literature on sporadic intraabdominal desmoids and inflammation as we investigated the mutational and epigenetic makeup of a case of multiple synchronous mesenterial desmoids occurring after necrotizing pancreatitis. A 62-year-old man had four mesenteric masses up to 4.8 cm diameter detected on CT eighteen months after laparotomy for peripancreatic collections from necrotizing pancreatitis. All tumors were excised and diagnosed as mesenteric desmoids. DNA from peripheral blood was tested for a multigene panel. The tumour DNA was screened for three most frequent β-catenin gene mutations T41A, S45F and S45P. Expression levels of miR-21-3p and miR-197-3-p were compared between the desmoid tumors and other wild-type sporadic desmoids. The T41A CTNNB1 mutation was present in all four desmoid tumors. miR-21-3p and miR-197-3p were respectively upregulated and down-regulated in the mutated sporadic mesenteric desmoids, with respect to wild-type lesions. The patient is free from recurrence 34 months post-surgery. The literature review did not show similar studies. To our knowledge, this is the first study to interrogate genetic and epigenetic signature of multiple intraabdominal desmoids to investigate potential association with abdominal inflammation following surgery for necrotizing pancreatitis. We found mutational and epigenetic features that hint at potential activation of inflammation pathways within the desmoid tumor.

## Introduction

Desmoid tumours (DT) are a rare form of mesenchymal proliferative disease characterized by monoclonal fibroblastic proliferation arising from deep soft tissue and mesenchymal stem cell progenitors [1]. The estimated incidence ranges from 5 to 6 cases per million per year, with a peak age of 30–40 years and a female predominance [2]. A variety of anatomic DT locations have been described including extremities, abdominal wall, head and neck, chest wall and intra-abdominal locations [3, 4].

Aside the apparent absence of metastatic potential, DT show tendency toward infiltrative growth and local recurrence [5, 6]; in case of intrabdominal lesions, this may lead to gastrointestinal complications as bowel obstruction and ischemia [7].

DT develop usually as solitary lesions, whereas multifocal DT occur on rare occasions, comprising less than 5% of all cases [8].

Intra-abdominal DT are rare and may often represent genetically determined lesions, occurring as an extracolonic manifestation of familial adenomatous polyposis (FAP), especially Gardner syndrome, which are associated with a germline mutation in the APC gene [9]. Sporadic intra-abdominal DT are uncommonly described as result of mutations in the CTNNB1 gene, encoding for β catenin involved in cellular adhesion and cellular transcription [10].

Heterogeneity in clinical and biological behavior and absence of histological/biological markers represent the two hallmarks of DT, which sometimes can lead to misdiagnosis and consequently to unsuitable therapeutic options [11]. Aside classic clinical prognostic factors as size and grading, a better understanding of the genetic fingerprint of desmoids may be more reliable in stratifying disease aggressiveness; studies have begun to identify gene expression profiles that may better predict tumor biology [12].

It is also known that history of previous trauma may precede the occurrence of sporadic desmoid tumors, and although no specific pathogentic mechanism has been elucidated, involvement of inflammation pathways has been evidenced in relation to the occurrence of DT.

We reviewed the recent literature addressing the pathogenesis and treatment of sporadic intraabdominal DT and investigated the genetic and epigenetic makeup of a rare case of multiple, synchronous sporadic intraabdominal DT in a patient who previously underwent open necrosectomy for infected pancreatic necrosis.

## Patient and methods

### Patient history

**Fig. 1:**
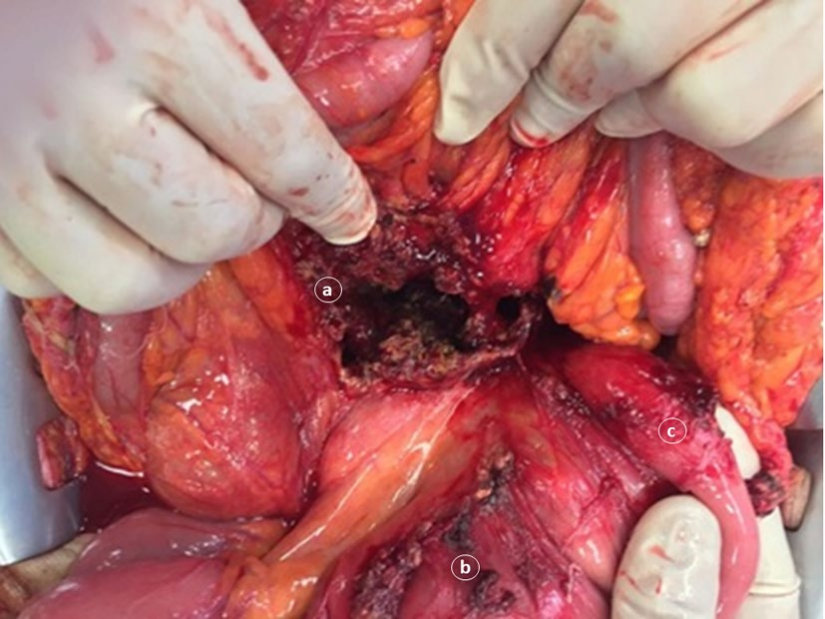
Laparotomy for necrotizing pancreatitis eighteen months before admission for desmoid tumors; pancreatic necrosis eroding into the transverse mesocolon (**a**), partly walled off by the mesentery (**b**) of the first jejunal loop (**c**)

A 62-year-old man was admitted to our Unit for elective treatment of symptomatic incisional hernia. His past medical history revealed benign prostate hyperplasia, cholecystectomy and emergency open necrosectomy for infected pancreatic necrosis, performed at our Institution eighteen months before the current admission (Fig. 1). Family history was relevant for death from neoplasm of the patient′s sister (liver) and of two brothers (brain, colonic), only one of whom at an adult age.

**Fig. 2:**
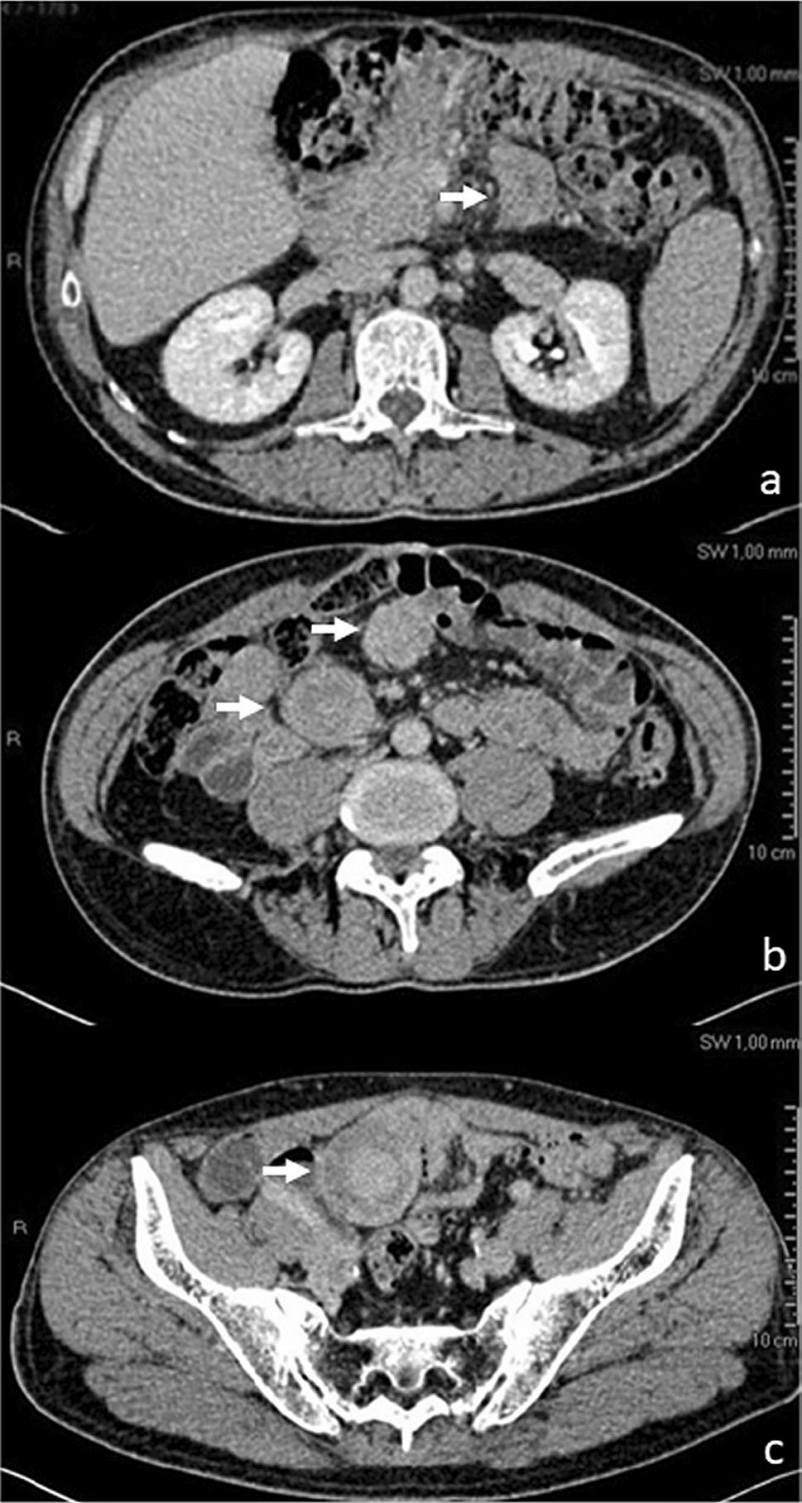
Contrast CT scan eighteen months after laparotomy for walled off pancreatic necrosis, showing four discrete intraabdominal masses (arrows), ranging from 1.6 to 4.8 cm diameter, isoattenuating to hyperattenuating to muscle

Clinical examination confirmed midline abdominal incisional hernia and abdominal distention. CT-scan of abdomen, performed to study the fascial defect, unexpectedly revealed four peritoneal solid lesions located in proximity of the branches of the superior mesenteric artery, with a diameter of 1.6, 2.8, 4.5, 4.8, respectively (Fig. 2a–c). Comparison with CT scan performed ten months before showed that these masses were not previously present, while their current position matched topographically that of former pancreatic necrosis, as it had been observed at the time of the previous laparotomy for necrotizing pancreatitis [13]. Although diagnosis was uncertain on the basis of imaging alone, the lesions were deemed to be compatible with GIST tumors on imaging, and surgical exploration was planned.

**Fig. 3:**
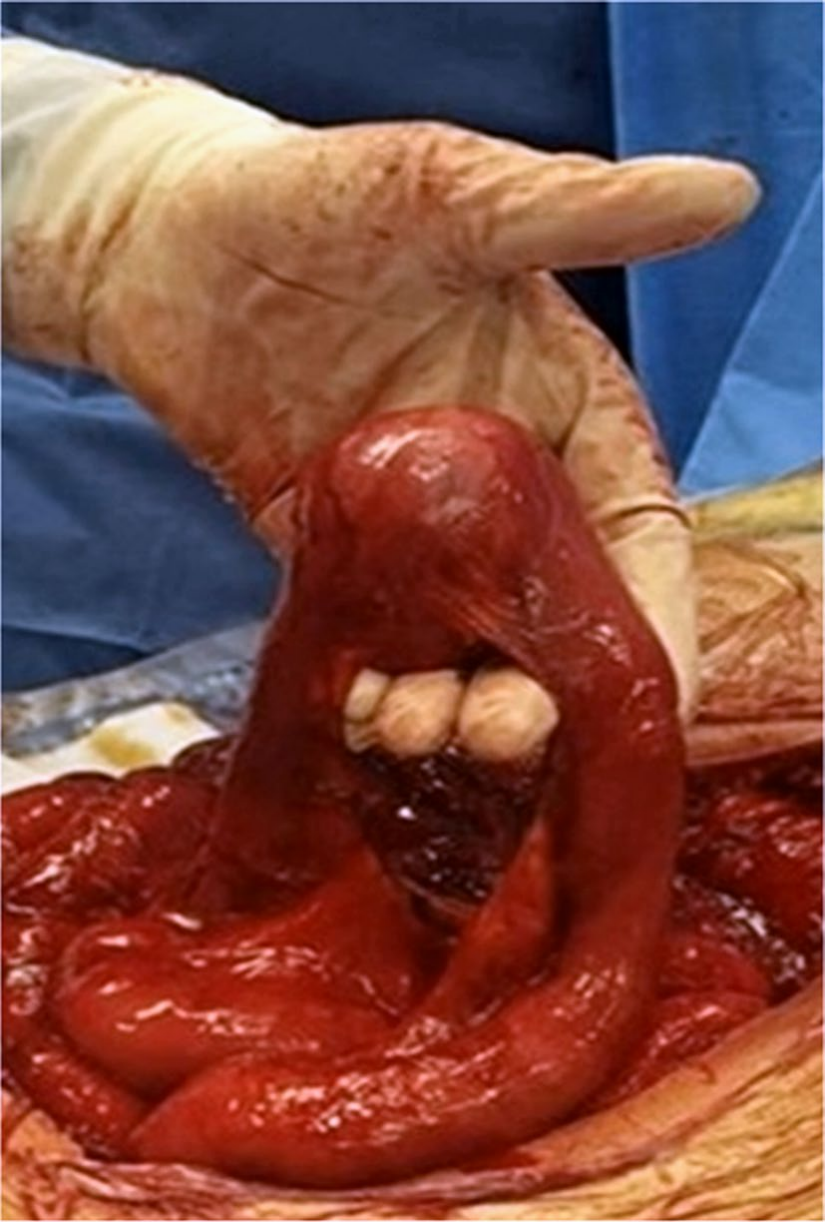
One of the three mesenteric masses resected at first surgical approach, lifted up and showing no cleavage from the small bowel. Working diagnosis included GIST

At laparotomy, two lesions were found in the vicinity of the cecum, one at the distal jejunum and a further one at the proximal jejunum, close to the origin of superior mesenteric artery (SMA). After extensive adhesiolysis, resection of the two pericecal masses and of the distal jejunal lesion was performed *in bloc* with the adjacent ileum and ascending colon (Fig. 3) followed by a jejuno-ileal anastomosis and ileo-colonic anastomosis respectively. Intraoperative frozen section showed highly dense collagen matrix and low cellular density of lesions and could not exclude malignancy. Removal of the mass located near the origin of the SMA was withheld to obtain definitive pathology result of the other similarly-appearing masses first, while limiting further operative risks. Incisional hernia was repaired with a direct suture technique. The patient recovered with no complications.

**Fig. 4:**
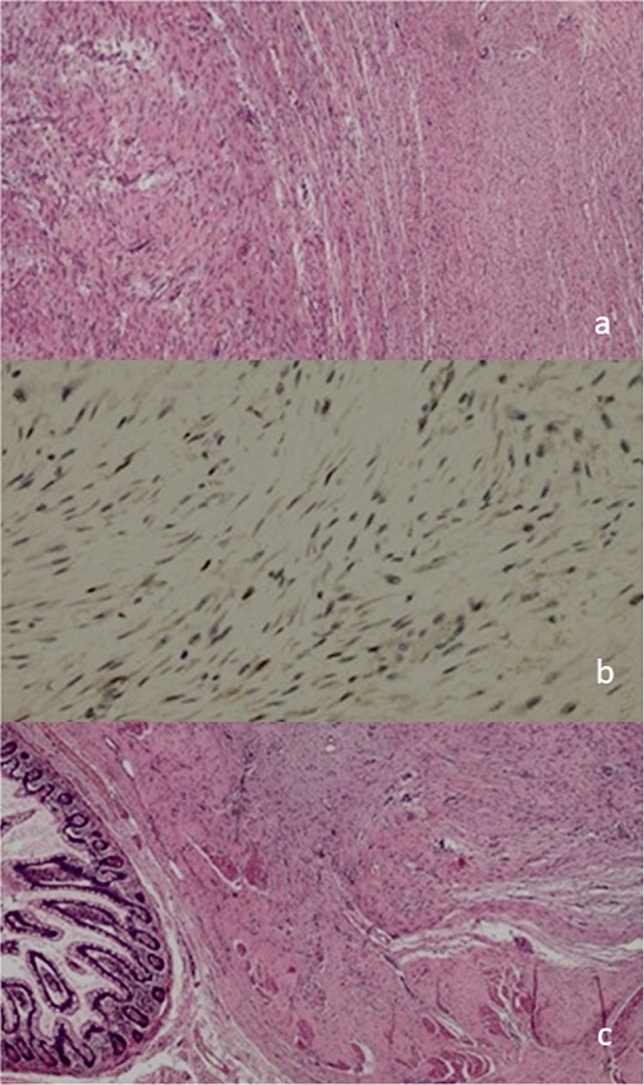
Pathology of the three desmoid tumors resected: **a** Spindle cell proliferation arranged in long fascicle; **b** nuclear positivity for beta-catenin; **c** assive infiltration of ileal wall

Definitive pathology revealed spindle cell proliferation arranged in long fascicles, showing nuclear positivity for b-catenin and infiltration of the duodenal wall, diagnosing all lesions as desmoid-type fibromatosis (Fig. 4). Ki 67 was < 5%. Postoperative colonoscopy showed no evidence of FAP syndrome. Follow-up of the remaining mass was initiated.

**Fig. 5:**
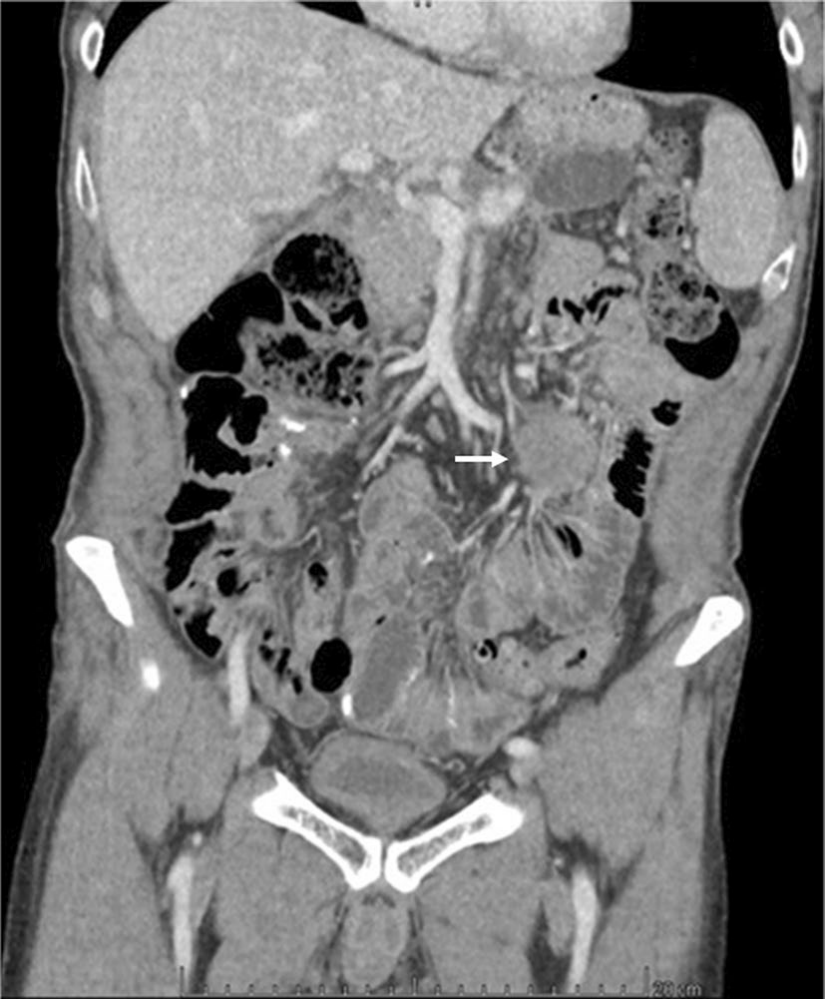
Coronal scan of contrast CT three months after resection of three intraabdominal sporadic desmoids: the patient was symptomatic for abdominal pain; the fourth lesion, located at the mesentery of the first jejunal loop, had increased in volume with respect to previous control, and there were signs of vascular and bowel compression

**Fig. 6:**
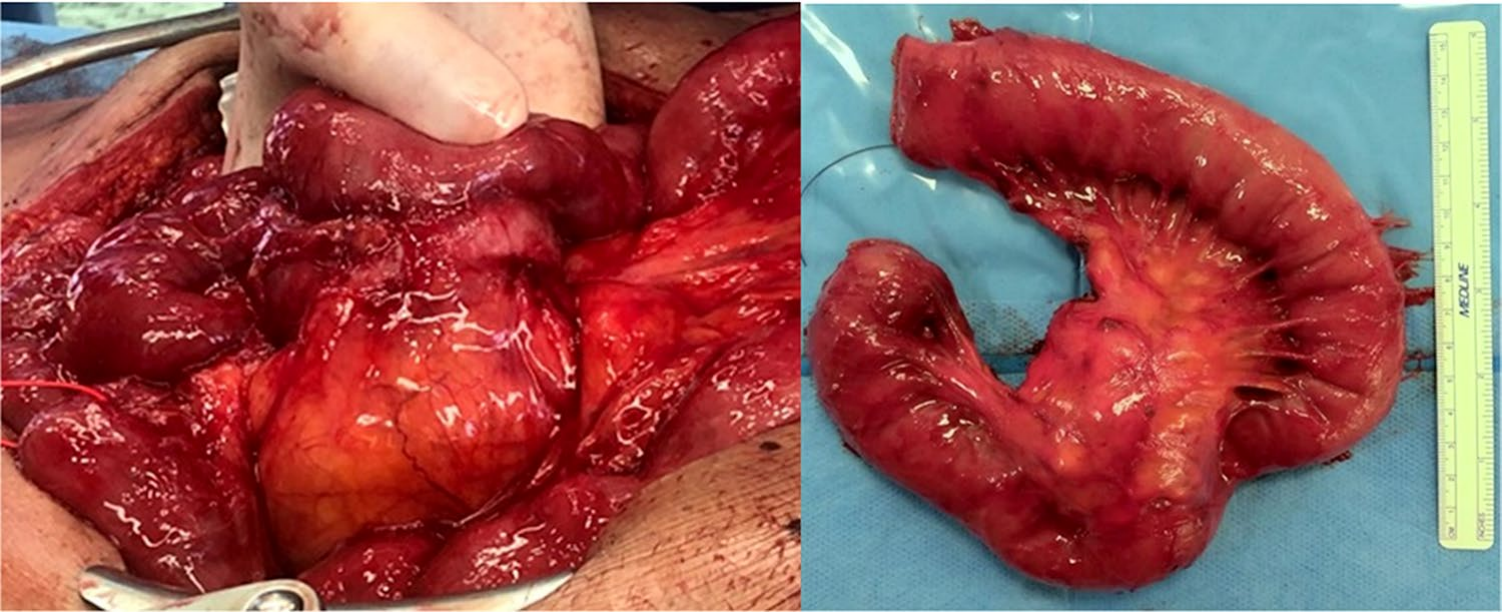
Intraoperative **a** aspect of the fourth desmoid tumor and specimen of in bloc resection with the first jejunal loop

CT scan performed three months post-excision showed a volumetric increase of the residual lesion, accompanied by anorexia and worsening gastro-oesophageal reflux, indicating surgical removal (Fig. 5). Resection of the remaining mass was performed in bloc with the adherent loop of proximal jejunum, after microsurgical dissection from the early branches of the SMA (Fig. 6). Postoperative course was uneventful, and the patient was discharged on the 7th postoperative day. The final histopathological analysis confirmed desmoid-type fibromatosis. The patient is currently on follow-up, with no evidence of recurrence on contrast CT scan 34 months after excision of desmoids.

### Genetic and epigenetic studies

#### Sample collection

The study was carried out on formalin-fixed paraffin-embedded (FFPE) tumor specimens of the DTs obtained by the Pathology Department. Consecutive FFPE tissue Sects. (4-μm thick) from the same block of each sample were cut and treated for immunohistochemical analysis, DNA mutations and miRNAs. DNA was isolated from the 4 desmoid tumors and from the pancreatic tissue that was found in the specimens of peripancreatic collections removed at the time of the previous surgery for acute necrotizing pancreatitis. DNA isolated from peripheral blood mononuclear cells (PBMNCs) was used to perform next generation sequencing (NGS).

#### Genetic analysis

NGS was executed using a multigene panel which included the following cancer predisposing genes: APC, ATM, BARD1, BMPR1A, BRIP1, CDH1, CDK4, CDKN2A, CHEK2, EPCAM, MLH1, MRE11A, MSH2, MSH6, MUTYH, NPN, PALB2, PMS2, PTEN, RAD50, RAD51C, RAD51D, SMAD4, STK11, TP53.

The DNA of the sporadic DTs was screened to evaluate the presence of the three most frequent mutations of the *CNTTB1* gene (*c.121A* > *G* (p.T41A), *c.134C* > *T* (p.S45F) and *c.133 T* > *C* (p.S45P)).

The amplification of the CTNNB1 exon 3 gene was performed using the following primers: CTNNB1e3F 5′- CAATCTACTAATGCTAATACTGTTTC-3′, and CTNNB1e3R 5′-CATTCTGACTTTCAGTAAGGCAATG-3′. The PCR products were Sanger sequenced on an ABI 3100 automatic capillary sequencer.

The *CNTTB1* gene mutations were detected by three qBiomarker Somatic Mutation PCR Assay kits (catalog # SMPH003987A, # SMPH003953A and # SMPH003970A for 121A > G, 134C > T and 133 T > C mutation, respectively) (Qiagen) according to manufacturer's protocol.

#### RNA isolation

Total RNA was isolated from FFPE tissue samples by miRNeasy FFPE Kit (Qiagen, Milan, Italy). All procedures were performed according to manufacturer's protocols. The yield and quality of RNA was determined by Nanodrop ND-1000 Spectrophotometer (Thermo Fisher Scientific, Wilmington, DE, USA. All RNA samples were stored at − 80 °C until analysis.

#### RT-qPCR for miRNA expression

Expression levels of miR-21-3p and miR-197–3-p [14], 15 were compared between the four desmoid tumors and nine “Wild-type” sporadic desmoid lesions (not showing CTNNB1 exon 3 mutation). miR-21-3p and miR-197-3p were selected as they appear involved in the inflammatory process and pro-fibrotic effect via their mRNA targets [16], 17, 18.

The RT-qPCR procedures were previously described [15]. All PCR tests were carried out in triplicate using cDNA synthesized from the same batch and miRNA expression was calculated by ΔCt method using U6 for normalization.

Informed consent was obtained from the patient at the time of surgery. This study was performed in line with the principles of the Declaration of Helsinki. Approval was granted by the Ethics Committee of of University of Bari, Italy (n. 6559/20).

## Results

### Pathological features and immunohistochemistry

The four desmoids presented with a circumscribed surface. Cut section showed grey-white color and infiltrative border on mesenteric fat and intestinal wall. The main pattern consisted of long fascicles, composed of uniform and slender fibroblast/myofibroblast with low cellular density. The cells had pale-staining nuclei with inconspicuous nucleoli without nuclear hypercromasia or cytological atypia. Mitotic count was very low (under 1 for 50 HPF). In each of the lesions, immunochemistry staining was positive for nuclear β-catenin in about 30% of the tumor, while it was negative for CD117, DOG1, S100, SM Actin, Desmin and CD34; proliferation index Ki 67 was < 5%.

## Genetic analysis

Next generation sequencing analysis revealed no pathogenic variants in any of 25 genes included in the multigene panel used. The mutation analysis of the CTNNB1 exon 3 revealed the presence of the same c.121A > G (p.T41A) mutation on all four desmoid tumors.

## Micro RNA analysis

miR-21-3p was upregulated in the mutated, sporadic mesenteric desmoids than in Wt group, whereas miR-197-3p was down-regulated in the mutated lesions with respect to Wt tumors.

## Discussion

To our knowledge, this is the first study to interrogate the specific genetic and epigenetic signature of multiple, synchronous intraabdominal desmoids to investigate their potential association with abdominal inflammation following surgery for necrotizing pancreatitis. The fact that four distinct mesenteric DT matched the location of previous peripancreatic collections occurring within the relatively short one-and-a-half year time interval after laparotomy favored further research into the mutational and epigenetic makeup of these neoplasms. DT is an uncommon stromal tumor that can develop sporadically or in association with hereditary syndromes.

Approximately 10–15% of DT are associated with germline mutations of adenomatous polyposis coli (APC), a tumor suppressor gene that normally regulates the β-catenin/Wnt growth factor pathway. In general, deregulation of the Wnt pathway results in aberrant proliferation, migration, and differentiation of cells into human cancers [19], 20. In cases of Familial Adenomatous Polyposis (FAP), a truncated APC protein with low affinity to β-catenin is created, resulting in the nuclear accumulation of β-catenin and consequently, overexpression of its target genes [4]. This provides different syndromic associations, with sex ratio close to one: DT associated with FAP, Turcot syndrome or DT as part of Gardner syndrome [10], 12, 13–15.

Most DT (85–90%) however are sporadic and are associated with somatic mutations of CTNNB1, a proto-oncogene which regulates cell adhesion and cell transcription in up to 91% of these cases [21, 22, 23, 24, 25, 26, 27]. β-Catenin, encoded by CTNNB1, is found in the cytoplasm and its activation leads to nuclear translocation [28]. In DT, there is an abnormal stabilization and nuclear accumulation of β-catenin. β-catenin binds transducin beta-like protein 1 (TBL1/TBLR1), and this complex stimulates the expression of several Wnt/APC/β-catenin pathway target genes, including proliferative factors, such as S100A4 or collagen triple helix repeat containing-1 (CTHRC1), identified as cancer-related proteins[29, 30, 31]. In sporadic DT, median age at diagnosis is approximately 40 and there is female predominance (M:F 1:2), [32]. The incidence of intra-abdominal sporadic DT is very low (5%); of these, only 10 cases of pancreatic DT have been published in the literature over the last 30 years [1, 5–7, 33–35].

Although CTNNB1 and APC mutations are mutually exclusive, they affect the same endpoint, the Wnt/ β-catenin pathway[3] suggesting a near universal relationship between desmoid tumors and Wnt signaling [4]: with deep sequencing 95% of desmoids may be found that have mutations affecting the Wnt/APC/β-catenin signaling pathway, and whole-exome sequencing and genomic analysis identified no other recurrent alteration [36, 19, 37, 10].

In sporadic DT three different β-catenin CTNNB1point mutations have been described in major mutation hotspots: T41A (approximately 55%), S45F (approximately 35%), and S45P (approximately 10%) [36]. These hotspots affect the site of APC/β-catenin interaction and alter the ability of APC to bind β-catenin and drive its degradation by the proteasome (inactivation of β -catenin destruction complex), leading to β-catenin nuclear accumulation [38, 39, 28]. DT carrying T41A or S45F mutations and Wild-Type (WT) β-catenin appear as two distinct molecular subgroups with regard to β-catenin stability, α-catenin affinity, and gene expression profiling [12]. There is also evidence that a different inflammation signature characterized S45F- and T41A-mutated cases, suggesting a specific inflammation setting mediated by the type of β-catenin mutation [40].

The clinical presentation of sporadic DT is based on site of origin: abdominal wall fibromatosis, extra-abdominal fibromatosis occurring in the head, neck or extremities, and intraabdominal fibromatosis that develops like mesenteric DT-type fibromatosis. DT have no known metastatic potential; rather, these tumors tend to be locally aggressive and invade adjacent structures with a morbidity around 10%. The patient is usually asymptomatic or symptoms related to the mass effect exerted by the tumor and local invasion [21].

Mesenteric-type DT are increasingly recognized as different clinical, histological and molecular entities from desmoids localized in the abdominal wall and extra-abdominal. They are frequently misdiagnosed due to partial clinical overlap with GIST tumors and liposarcomas [41–44]. Molecular typing of sporadic intraabdominal DT demonstrates that the CTNNB1 T41A mutation is largely prevalent (80.4% of cases) [15, 23–26, 40]. Given the high frequency of mutations of the β-catenin gene that characterizes sporadic DT, the analysis of the mutational status of CTNNB1 has been suggested as a useful tool in the differential diagnosis with other soft tumors [15, 45, 46].

In the case presented here, all four synchronous DT were characterized by the same CTNNB1 mutation, confirming that the T41A is a mutation hotspot in mesenteric DT and supporting the hypothesis that such mutation is selected in the mesenteric localization for its unique and site-specific selective advantage [12]. T41A mutation-bearing desmoids also show overexpression of anti-inflammatory markers associated with antitumor immunity compared with S45 [40]. This peculiar inflammatory microenvironment is in line with the hypothesis that β-catenin signaling activation underpinning DT growth results in T-cell exclusion favoring the immune evasion [47]. Further, the T41A mutation appears to confer a lower risk of recurrence compared to the S45F mutation [23]. Thus, molecular analyses may be an important aid for an accurate diagnosis and a precise evaluation of recurrence risks.

Molecular characterization of multiple synchronous mesenteric DTs is instead underreported in literature. By combining genetic and epigenetic analysis, our findings extend those of ourselves and others [15, 48, 49]. Previous microarray analysis respectively conducted in APC and CTNNB1-mutated intraabdominal DT revealed a great number of miRNAs differently expressed in these two tumor types compared to WT controls, suggesting a deep molecular heterogeneity among them, despite their histological similarity; about 90% of dysregulated miRNAs were present in sporadic DTs and were linked to several biological functions, as cell proliferation and differentiation, response to the inflammatory process and tumor growth [15].

Previous RT-qPCR data from our Institution showed that miR-21-3p was significantly altered in sporadic mutated DT, compared to WT. The over-expression of miR-21-3p was associated to high levels of nuclear β-catenin protein in mutated DT [15].

Related to multiple cancers [50, 51], miR-21 targets and inhibits the tumor-related genes of programmed cell death 4 (PDCD4), and phosphatase and tensin homolog (PTEN) [52]; it is involved in tumor angiogenesis, which is closely related to tumor cell proliferation, invasion, and drug resistance [53, 54]. We found that miR-21 was upregulated in the four synchronous desmoids showing significant nuclear positivity for β-catenin, in line with experimental evidence that overexpressed miR-21-5p associated with mutated CTNNB1 promoted increased phosphorylation of β-catenin and nuclear translocation in colorectal cancer cell lines [55].

Consistently also with previous data from our Institution, this study also found miR-197 to be downregulated compared to wild-type DT.

Transcribed from the genomic region of chromosome 1p13.3, miR-197 has been found to be significantly dysregulated in a wide range of cancers including lung [56], breast [57], ovarian [58], colorectal [59], thyroid [17] and prostate cancer [60], head and neck [61] and hepatocellular carcinoma(HCC) [62].

Taken together, the functions of miR-197 in cell proliferation are not well known. In particular, miR-197 showed a double identity for apoptosis in diverse cancers [18]: miR-197 decreased apoptosis in lung cancer through inhibiting the expression of p53 [63]; in contrast, overexpressed miR-197 was accompanied by induction of apoptosis in multiple myeloma cells [64]. MiR‐197 was also shown to induce epithelial–mesenchymal transition in pancreatic cancer cells by targeting p120 catenin, a crucial process developing in tumor invasion and metastasis [65]. Moreover, research on lung cancer showed evidence that miR-197 may be involved in tumor immune regulation [66].

Dysregulated miR-197 in various cancers received sophisticated regulatory [18]: IL-6 appeared to stimulate and increase the levels of p-SATA3 and STAT3 protein, which inhibited the expression of miR-197 and thus contributed to oncogenesis via promoting cell proliferation, preventing apoptosis [67].

The inflammatory pathway interferes with mature miR-197-3p biosynthesis [30] in the HCC cell model through increased SERPINA3 levels that were associated to the acute and chronic inflammation [31]; it has been hypothesized that over-expression of miR-21-3p and down-expression of miR-197-3p targeting L1CAM, SERPINA3 and TSPAN3, respectively, could induce or maintain the inflammatory process in mutated DT, although the physiological function of the SERPINA3 protein in sporadic DT is unknown [15]. However, it has also been noted that CTNNB1 gene produces a phosphorylated form of the β-catenin that evades the miRNA regulatory effect [15].

The pathogenesis of DTs is not entirely clear: aside genetic predisposition, development of desmoids is most commonly associated with pregnancy, hormonal exposure [68] and physical factors such as trauma including previous motor vehicle accidents [69] and/or surgery [70–72].

While it is difficult to prove the relationship between trauma and development of this disease, the incidence of trauma preceding the development of DT is greater than that reported for other soft tissue tumors [73]. It has been proposed that this association may in fact be the result of aberrant proliferation after a traumatic insult, with an underlying over-activation of the molecular signaling pathway for normal wound healing [74].

Studies also demonstrated a possible correlation with tissue repair processes through the evidence of elevated β-catenin levels in fibroblasts during the proliferative phase of healing [75]: in mouse models β‐catenin was shown to be an important part of normal cutaneous wound healing and stabilization of β‐catenin alone was sufficient to cause aggressive fibromatosis [75]. A possible derivation of DT was also described from multipotent mesenchymal stromal cells (MSCs) acquiring mutations in the APC gene with increase of β-catenin levels during a defective wound healing; the presence of MSCs has been shown within human DT, which could explain the correlation between surgery and DT localization [76].

As for the risk of developing multifocal DT, while research is limited due to the rare nature of this disease, it appears that chronic inflammation and repetitive trauma may be considered a risk factor [77].

We observed multifocal DT in the areas where peripancreatic collections from necrotizing pancreatitis were previously present. As beta-catenin plays an important role in normal homeostasis of the pancreas, a seeding effect, with multiple DTs possibly originated from the site of a sustained and chronic inflammation in the pancreas, cannot be formally excluded due the low sensitivity of genetic analyses. It is known that beta-catenin is an important component of the epithelial adherens junction together with E-cadherin, cytoplasmic p120-catenins and α-catenins: dynamic changes of the adherens junction structure appear to underlie the initiation and progression of acute pancreatitis (AP) as well as the following repair mechanisms [78]. Also, isolate reports of DT occurring shortly after surgery for pancreatic carcinoma are all of mesenteric DT [79]. However, despite its important role in normal pancreas homeostasis, such a mechanism has never been demonstrated. Moreover, the CTNNB1 gene is rarely mutated in pancreatic cancer irrespective of the histologic type [80]. Thus, based on this report and the literature, it appears that mesenteric DT are likely consequences of surgical procedures on the pancreas [34, 35].

DT is typically suspected on imaging studies, with a pathological specimen required for definitive diagnosis. Imaging features depend on degree of fibrosis, vascularity, fibroblast proliferation and collagen content [81] Computed tomography demonstrates a well-defined mass with variable enhancement pattern, typically isoattenuating to hyperattenuating to muscle on contrast-enhanced imaging with minimal washout [82–84]. The mesenteric desmoids that we studied were dense masses on contrast-enhanced CT. At magnetic resonance DT show low signal intensity compared to muscle on T1-weighted images, while T2 signal is variable, depending on presence of cystic content [81]. [81]. On PET/CT scan appearances are variable, with previous studies describing larger masses as moderately hypermetabolic and smaller lesions hypometabolic; uptake values have been reported between 3.1 and 4.1 [83, 85]. In the absence of known primary malignancy, the differential diagnosis on imaging is typically other soft tissue malignancies such as leiomyosarcoma including GIST [41, 42], rhabdomyosarcoma, synovial sarcoma, fibrosarcoma, liposarcoma, and fibrous histiocytoma [43]. Lymphoma is always a possibility in the mesentery, with metastatic disease also to be considered [86]. In the case that we treated, possible lymphoma, GIST and metastatic lesions from unknown primary were also considered before surgery.

As the natural history of desmoids has become better understood, it is increasingly evident that some tumors will not grow and may even spontaneously regress, sparing patients the morbidity of more aggressive therapy. Current guidelines favor conservative management of DT with observation/surveillance, as DT have been shown to be slow growing or halt spontaneously in 30- 50% of tumors [87, 88]. Spontaneous regressions of DT may owe to the immunologic environment of the host, although the role of immunity in the course of the disease is currently under study [88]. However, the majority of cases documenting spontaneous regression with conservative management were extra-abdominal [5, 89]. A consensus treatment algorithm, proposed by the Soft Tissue and Bone Sarcoma Group (STBSG), Sarcoma Patients EuroNet (SPAEN) and European Organization for Research and Treatment of Cancer (EORTC), acknowledged that while spontaneous regression had been observed at all anatomical sites of desmoid tumors, surgery remains the main treatment for progressive and operable intra-abdominal tumors [88]. A step wise treatment plan for sporadic DT approved by the French and Italian Sarcoma group also supported an initial waiting period, but acknowledged the need for immediate medical or surgical intervention considering location (head, neck, pelvis, or intraabdominal cavity), size, and symptoms presented by patient [5]. Our patient had multiple, intra-abdominal symptomatic tumors with documented mass effect on intra-abdominal organs and bowel in addition to worsening abdominal symptoms over a short time frame: this indicated an aggressive growth pattern and need for surgical intervention rather than conservative management. Further risk of vessel compression also indicated surgical intervention. A multidisciplinary approach with detailed preoperative imaging and histologic analysis was used to assist with surgical planning.

Surgical resection with histologically negative margins has been the cornerstone of therapy for this disease [21]. However, there is controversy regarding the role of R0 resection in preventing recurrence [90]. While large retrospective studies have shown higher recurrence rates for patients with R1 margins compared to R0 margins, other studies have shown that margin status is a predictor of recurrence on univariate but not on multivariate analysis. Thus while R0 resection is preferred, recurrence rates following R0 and R1 resection may be similar [21]. It is now common to accept a microscopically positive margin after resection as recurrence rates may not be significantly affected., particularly when surgery is complemented with adjuvant therapy [88].

An example of adjuvant therapy is the tyrosine kinase inhibitor sorafenib. A recent randomized control trial demonstrated that patients with advanced(inoperable or symptomatic) DTFs that are treated with sorafenib have an improved 2-years progression-free survival rate compared to the placebo group (81 vs. 36%) [91]. Imatinib (800 mg PO daily) has also been successfully used in patients with DT. However, no definite mechanism or pathway of action is known [92].

Recurrence may contribute to the overall long-term survival of the patient. Retrospective studies showed that the median overall survival after diagnosis of primary disease was 10.2 years, and 6.9 years upon recurrence [93], and 10-year disease-free survival was 76% with primary disease and 59% with recurrent disease [94]. With regards to intra-abdominal tumors, each subsequent recurrence had an increased risk of compression and intimate association with vital organs and vessels limiting the resectability of the tumor.

Considering that sporadic DTs with the S45F mutation have a greater tendency for local recurrence, with increasing implementation of “watchful-waiting” for DT management, it will be important to determine whether mutation type predicts outcome for these patients [23, 95].

As our patient received treatment relatively recently, this case cannot provide adequate insight into DT prognosis and recurrence, so studies involving long term follow-up outcomes will be valuable in deepening the understanding of DT [96]. Also, this was an exploratory study and a limited number of target genes and micro-RNAs were considered. Despite these limitations, evidence from this study confirms previous research from our Institution: uniform signature of T41A mutation in synchronous, multiple mesenteric desmoids following surgical trauma and chronic inflammation, with upregulation of miRNA 21and downregulation of miR197, which is rarely investigated in DT. These results support further research to investigate the potential role of downregulated miR-197 in relation to T41A mutated sporadic mesenteric desmoids. Given the confirmation of previous findings, the pattern of dysregulation of miR-197-3p and miR-21-3p expression might also be investigated in combination with mutational analysis of the CTNNB1 gene as a potential, additional diagnostic tool in case of unclear pathological assignment of suspect intraabdominal DT.

## Conclusion

Sporadic intraabdominal desmoid type fibromatosis is known to potentially occur after trauma, including previous surgery. While management of desmoid tumors has made significant progress, knowledge of the underlying pathogenetic mechanism is still limited. We found that multiple mesenteric desmoids showing “clonal” mutational profile presented in a time interval and in abdominal locations that suggested relationship with previous surgery for necrotizing pancreatitis; building on previous research from our Institution, we also found epigenetic features that hint at activation of inflammation pathways within the desmoid tumor.

Our recent literature review shows that miR 197 in particular is emerging as a molecule both involved in chronic inflammation as well as in the progression of different solid tumors.

## Data Availability

Original Patient′s and accessory data supporting this study are available upon request from the Department of Biomedical Sciences and Human Oncology – Academic Unit of General Surgery “V. Bonomo”, Policlinico di Bari.
